# Balancing the medical and social needs of children during the COVID‐19 pandemic

**DOI:** 10.5694/mja2.51808

**Published:** 2022-12-05

**Authors:** Zoë Hyde

**Affiliations:** ^1^ Western Australian Centre for Health and Ageing University of Western Australia Perth WA

**Keywords:** COVID‐19

## Open access

Open access publishing facilitated by The University of Western Australia, as part of the Wiley ‐ The University of Western Australia agreement via the Council of Australian University Librarians.

## Competing interests

No relevant disclosures.

to the editor: In a recent Editorial,^1^ Grimwood and Chang cited a review of long COVID in children and adolescents,^2^ and wrote that symptoms are similar for those with and without evidence of severe acute respiratory syndrome coronavirus 2 (SARS‐CoV‐2) infection. But this is an inaccurate description of the review's findings. Cases were more likely to experience persistent symptoms than controls in the majority of studies reviewed.^2^ The difference in prevalence might be even greater than reported owing to the well documented underdetection of coronavirus disease 2019 (COVID‐19) in children,^3^ resulting in misclassification of cases as controls.

A growing body of evidence indicates children are more affected by COVID‐19 than initially thought. A recent US Centers for Disease Control and Prevention (CDC) analysis of 1.4 million children aged under 12 years and 1.7 million adolescents aged 12–17 years found increased rates of asthma, myocarditis and cardiomyopathy, cardiac dysrhythmias, diabetes, renal failure, venous thromboembolism, and coagulation disorders in children with laboratory‐confirmed COVID‐19 compared with children without COVID‐19. These increased risks (excluding asthma) were also experienced by adolescents with COVID‐19, who were additionally at increased risk of pulmonary embolism.^4^ Although uncommon or rare, such outcomes suggest children are not spared the cardiovascular and metabolic sequelae of COVID‐19.

Recent research using low field magnetic resonance imaging (MRI) revealed persistent pulmonary dysfunction in non‐hospitalised children and adolescents (mean age, 11 ± 3 years) who had recovered from COVID‐19 (*n* = 29) or had long COVID (*n* = 25). Despite all children having morphologically normal lungs (except for one recovered child), ventilation and perfusion (V/Q) matching was markedly lower in the recovered group (62 ± 19%) and the long COVID group (60 ± 20%) compared with nine healthy controls (81 ± 6%; mean age, 10 ± 3 years).^5^

Although the MRI study may be limited by selection bias (ie, children with greater symptomatology being more likely to participate), this and similar research indicate the health impact of paediatric COVID‐19 is greater than generally acknowledged. We do not know what the long term impact of SARS‐CoV‐2 infection might be, but the accumulating data are not encouraging.

Reinfection is common and SARS‐CoV‐2 spreads readily in schools in the absence of mitigation measures, such as the use of masks, portable HEPA air cleaners, and improved ventilation. Notably, better ventilation has wider benefits, including improved academic performance. A poorly ventilated classroom can be equivalent to a student skipping breakfast.^6^

The COVID‐19 pandemic is not over. Ongoing commitment to a public health strategy informed by the precautionary principle is required. This will deliver wide‐ranging social, economic and health benefits.
